# Use of Expert-Validated AI-Generated Forensic Images in Case-Based Learning and Clinical Reasoning Among Undergraduate Medical Students: A Two-Arm Educational Intervention Study

**DOI:** 10.7759/cureus.117464

**Published:** 2026-10-05

**Authors:** Priyanka Chahal, Abhishek Sharma, Arindam Chatterjee

**Affiliations:** 1 Forensic Medicine, Sudha Medical College and Hospital, Kota, IND; 2 Forensic Medicine, Kalyan Singh Government Medical College, Bulandshahr, IND; 3 Forensic Medicine, Mahatma Vidur Autonomous State Medical College, Bijnor, IND

**Keywords:** artificial intelligence, case-based learning, clinical reasoning, forensic medicine, generative artificial intelligence, medical edcucation

## Abstract

Background

Clinical reasoning is an essential competency in undergraduate medical education, while forensic medicine requires accurate visual recognition and interpretation of injury patterns. Case-based learning (CBL) provides a structured approach to developing higher-order reasoning skills. Generative AI offers a potential method for creating customized visual learning resources, although evidence regarding the educational value of AI-generated clinical and forensic images remains limited.

Objective

The study aimed to evaluate whether expert-validated AI-generated forensic images used as visual triggers within standardized CBL were associated with differences in clinical reasoning among Phase II and Phase III MBBS students compared with conventional forensic images.

Methods

This two-arm parallel-group non-randomized controlled educational intervention study enrolled and allocated 249 Phase II and Phase III MBBS students to AI-generated image or conventional-image groups. Images were generated using GPT-Image-1 (OpenAI, San Francisco, CA). The primary T0-T1 analysis included 227 students, while 218 students with complete T0-T1-T2 data contributed to the longitudinal analysis. The intervention group participated in standardized CBL sessions using expert-validated AI-generated forensic images, while the comparison group used conventional forensic images. Case scenarios, learning objectives, questioning sequence, faculty facilitation, session duration, and debriefing were standardized across groups. Clinical reasoning was the prespecified primary outcome. Secondary outcomes included knowledge, engagement, confidence, satisfaction, and short-term retention. The primary T0-T1 analysis included 227 students, while 218 students with complete T0-T1-T2 data contributed to the longitudinal analysis. Between-group comparisons were adjusted for baseline clinical reasoning and academic phase.

Results

Baseline characteristics were comparable between groups. The adjusted post-intervention clinical reasoning score was significantly higher in the AI-generated image group than in the conventional-image group (25.3 ± 3.9 vs 23.1 ± 4.2), with an adjusted mean difference of 2.4 points (95% CI, 1.4-3.4; p < 0.001; Cohen's d = 0.59). The AI-generated image group also demonstrated higher knowledge scores (25.1 ± 2.9 vs 23.7 ± 3.1; adjusted mean difference, 1.3; 95% CI, 0.5-2.1; p = 0.002), engagement (64.6 ± 6.2 vs 62.5 ± 7.2; p < 0.001), confidence (21.4 ± 2.3 vs 19.6 ± 2.8; p < 0.001), and satisfaction (22.8 ± 2.1 vs 20.5 ± 2.8; p < 0.001). At five to six weeks, clinical reasoning scores were 23.2 ± 4.1 in the AI-generated image group and 20.9 ± 4.3 in the conventional-image group, with a significant group-by-time interaction (p < 0.001). Knowledge also demonstrated a significant group-by-time interaction (p = 0.004).

Conclusion

This study found that students receiving expert-validated AI-generated forensic images as visual triggers within standardized CBL demonstrated greater improvement in measured clinical reasoning than students receiving conventional forensic images. These findings represent an association within a specific faculty-supervised educational setting and do not establish that AI-generated imagery alone caused the observed differences. AI-generated visual resources may be considered as an adjunct to competency-based forensic medicine education when incorporated within a structured pedagogical framework and subjected to expert validation.

## Introduction

Clinical reasoning is an essential competency in undergraduate medical education, requiring students to integrate clinical findings, contextual information, and medical knowledge to reach appropriate interpretations and decisions [1,2]. Case-based learning (CBL) provides a structured approach to developing these skills by encouraging students to analyse clinical information, apply knowledge, and engage in higher-order reasoning rather than simple factual recall [3,4].

Visual learning is particularly important in disciplines where recognition of morphological findings is central to clinical interpretation. In forensic medicine, students must identify and describe injuries, correlate their morphology with possible mechanisms, and apply these findings to medico-legal situations. Structured visual analysis has been shown to enhance observation and interpretation skills among medical students [5,6].

The rapid development of generative AI has created new opportunities for producing customized educational material, including clinical images and CBL resources. However, most existing applications of generative AI in medical education have focused on text generation, explanations, assessment, or conversational support, while evidence regarding the use of AI-generated images specifically to enhance clinical reasoning remains limited [7,8]. Concerns regarding anatomical accuracy and clinical realism also emphasize the need for expert validation and faculty supervision.

Therefore, this study evaluated whether the use of expert-validated AI-generated forensic images as visual triggers in standardized CBL improves clinical reasoning among undergraduate MBBS students compared with conventional forensic images. Secondary outcomes included knowledge, retention, engagement, confidence, and satisfaction.

## Materials and methods

Study design, setting, and participants

This two-arm parallel-group non-randomized controlled educational intervention study was planned among Phase II and Phase III MBBS students at Dr S.S. Tantia Medical College, Sri Ganganagar, India, from January to July 2025. The study was designed to evaluate whether the use of AI-generated clinical images as visual triggers during CBL was associated with differences in clinical reasoning compared with conventional forensic images.

The educational content was mapped to the Revised Competency Based Medical Education (CBME) Curriculum 2024 issued by the National Medical Commission (NMC) [9]. The principal competency domains selected for the study were mechanical injuries and medico-legal examination of injured persons. In particular, the intervention was aligned with FM5.1, which addresses the definition, description, classification, and medico-legal aspects of mechanical injuries, and FM14.1, which requires students to examine an injured person and prepare a medico-legal report in a simulated or supervised environment.

The minimum sample size was estimated for comparison of two independent groups using an anticipated standardized effect size of 0.50, a two-sided alpha level of 0.05, and 80% power. The resulting minimum sample size was 63 participants per group (126 participants in total). Allowing approximately 10% attrition increased the target minimum to approximately 70 participants per group. Three hundred eligible students were available across the two academic phases, with 150 students in each phase. Students who were enrolled in Phase II or Phase III MBBS, attended the scheduled teaching session, and provided informed consent were eligible for participation. Of the 300 eligible students, 33 were absent from the scheduled intervention, 12 declined participation, and six could not be reached despite attempts at contact. The remaining 249 eligible students were assigned to the two intervention groups using a systematic allocation procedure based on student roll numbers. The first eligible roll number from each batch was used as the starting point, and subsequent eligible students were assigned alternately to the AI-generated image and conventional-image groups. The allocation was performed by a faculty member. A formally generated random allocation sequence was not used, and allocation concealment was not implemented. The person responsible for enrollment and consent was aware of the allocation. Therefore, the study was considered a non-randomized controlled educational intervention. A total of 125 students were assigned to the AI-generated image group and 124 to the conventional-image group. After exclusion of participants with missing assessments, 109 students in each group had complete data at baseline assessment before the educational intervention (T0), immediate post-intervention assessment (T1), and five- to six-week retention assessment (T2) and were included in the longitudinal analysis. The primary T0-T1 analysis included 116 students in the AI-generated image group and 111 students in the conventional-image group (Figure 1). The study was reported in accordance with the principles of the Transparent Reporting of Evaluations with Nonrandomized Designs (TREND) framework [10].

**Figure 1:**
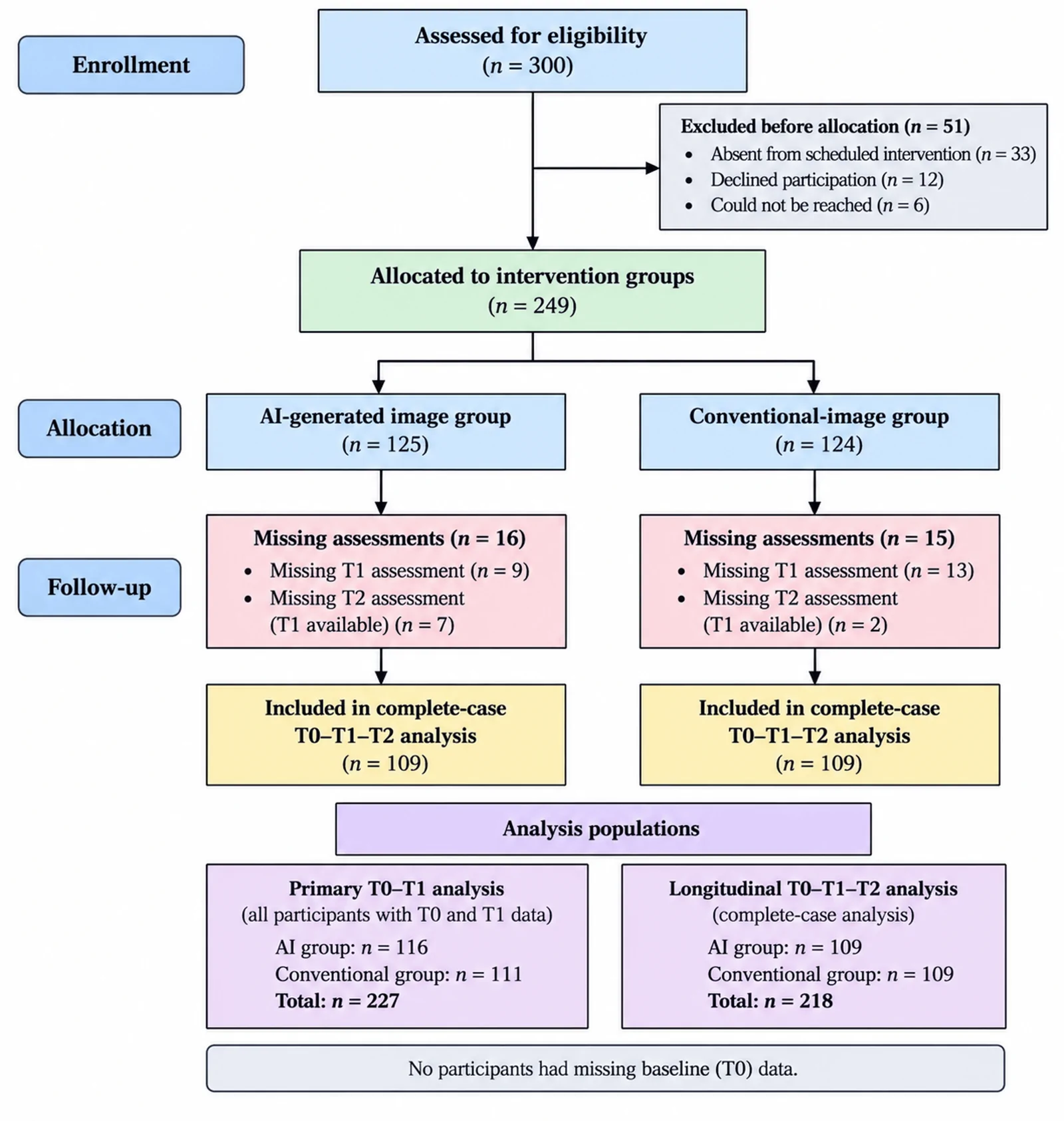
Participant flow diagram Abbreviations: n, number of participants; AI, artificial intelligence; T0, baseline assessment before the educational intervention; T1, immediate post-intervention assessment; T2, five- to six-week retention assessment. The primary T0–T1 analysis included all participants with available T0 and T1 data (n = 227), while the longitudinal T0–T1–T2 analysis included participants with complete data at all three time points (n = 218).

Ethical approval was obtained from the Institutional Ethics Committee of Dr. S.S. Tantia Medical College, Hospital & Research Centre (approval number: IEC/2025/061). Written informed consent was obtained from all participating students. Participation was voluntary, and students were informed that participation or nonparticipation would not influence their academic evaluation.

Development and implementation of the educational intervention

The intervention was developed using a competency-to-image-to-case framework. First, the selected NMC competencies were reviewed and converted into specific learning objectives. The learning objectives were designed to progress from visual recognition and description to interpretation, clinical correlation, medico-legal application, and recognition of the limitations of image-based inference.

The principal learning objectives were to enable students to identify common mechanical injuries, describe their morphology using appropriate forensic terminology, classify injuries according to their morphological characteristics, correlate visual findings with possible mechanisms, distinguish between commonly encountered injury patterns, identify findings that should be documented during medico-legal examination, formulate an appropriate description for a medico-legal report, and recognize the conclusions that could and could not reasonably be drawn from an image alone.

Additional visually demonstrable forensic findings, including selected thermal and asphyxial injury patterns, were incorporated where appropriate to broaden the application of image-based clinical reasoning. All additional content was mapped to the relevant institutional and NMC-CBME teaching objectives before inclusion.

The intervention content was developed around standardized case scenarios. For each case, the faculty first defined the expected forensic finding, anatomical site, relevant clinical context, learning objective, and essential visual characteristics. These specifications were then converted into standardized prompts for ChatGPT (OpenAI, San Francisco, CA, USA). The AI tool was used solely to generate the visual stimulus; it was not used to teach the students, provide explanations, answer questions, assess student responses, or determine the correct diagnosis. 

A standardized prompt format was used. Prompts specified the intended forensic finding, anatomical location, clinical context, educational purpose, and required degree of anatomical and medical realism. Images were requested without diagnostic labels, arrows, text, or captions so that students were required to interpret the visual information independently. Images were generated using GPT-Image-1 accessed through ChatGPT in March 2025. Images were generated for each learning objective, and prompts were refined, or images were regenerated when an initial output showed anatomical inaccuracies, inappropriate morphology, inadequate visual clarity, or other features inconsistent with the predefined educational requirements. Generation settings were not independently manipulated, and image selection was based on predefined educational and visual criteria rather than technical generation parameters. The faculty subsequently screened the generated images for anatomical plausibility, forensic accuracy, clinical realism, visual clarity, relevance to the intended learning objective, and appropriateness for undergraduate medical education. Images containing inaccurate anatomy, misleading morphology, implausible injury patterns, excessive visual distortion, or other educationally important errors were rejected and regenerated. A representative generation prompt is provided in the Appendices.

The validation process therefore involved an initial faculty screening for anatomical, forensic, clinical, and visual suitability followed by formal content validation using the predefined Item-Level Content Validity Index (I-CVI) criterion. A total of 100 AI-generated images were initially developed for the educational intervention. A sample AI-generated image is shown in Figure 2.

**Figure 2:**
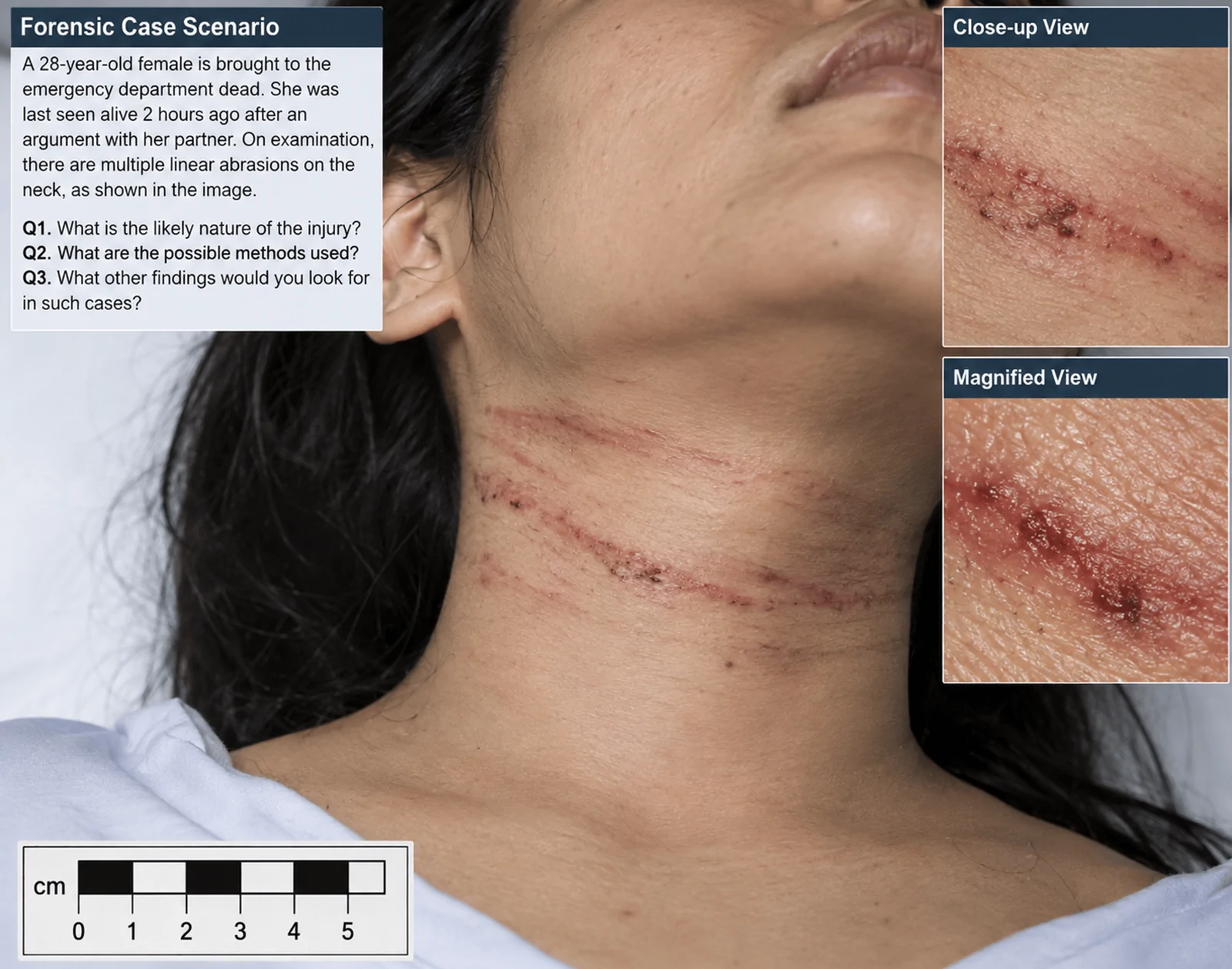
Sample of the AI-generated image dataset Image generated using GPT-Image-1 accessed through ChatGPT (OpenAI, San Francisco, CA, USA).

The images were independently evaluated by five faculty experts from the Department of Forensic Medicine for overall educational relevance and appropriateness using a four-point rating scale, where 1 indicated not relevant, 2 indicated needs major revision, 3 indicated relevant, and 4 indicated highly relevant. The I-CVI was calculated for each image as the proportion of experts assigning a rating of 3 or 4. With five experts, the possible I-CVI values were 0.00, 0.20, 0.40, 0.60, 0.80, and 1.00. Thus, an I-CVI of ≥0.80 required at least four of the five experts to rate an image as relevant or highly relevant. The formal I-CVI assessment represented educational relevance and appropriateness, whereas clinical and forensic accuracy were assessed separately during the faculty screening process based on anatomical plausibility, forensic accuracy, clinical realism, visual clarity, and suitability for undergraduate education. An I-CVI of ≥0.80 was predefined as the criterion for acceptable content validity. Images that did not meet this criterion were excluded from the final image set. Following expert evaluation, 62 of the 100 AI-generated images (62%) met the predefined validity criterion and were retained for use in the educational intervention.

The comparison intervention used conventional forensic photographs or established standard educational images representing the same findings and topics. The conventional images were selected from the departmental de-identified teaching image repository with appropriate permission and confidentiality safeguards. The AI-image and conventional-image groups received identical learning objectives, case scenarios, faculty facilitation, duration of teaching, sequence of questioning, and post-session explanations. Thus, the principal difference between the groups was the source of the visual stimulus. A total of 232 conventional images were initially identified. These were available in their existing digital formats, predominantly JPEG, without a predefined resolution criterion. The images were independently evaluated by the same five faculty experts from the Department of Forensic Medicine using the same four-point relevance scale used for the AI-generated images (1 = not relevant, 2 = needs major revision, 3 = relevant, and 4 = highly relevant). Selection focused on relevance to the forensic finding, anatomical site, learning objective, and educational purpose. Faculty members then matched the shortlisted images to the corresponding findings and learning objectives represented by the 62 AI-generated images, resulting in a final set of 62 conventional images. Formal matching for visual complexity, realism, image quality, diagnostic salience, or difficulty was not performed.

Each CBL session followed a standardized sequence. Students were initially shown the image without a diagnosis or explanatory labels and were asked to observe and describe the visible findings. Progressive clinical and medico-legal information was then provided, followed by structured questions requiring students to identify the finding, describe its morphology, interpret possible mechanisms, correlate the finding with the case history, determine what additional information would be required, and formulate an appropriate medico-legal interpretation. The session concluded with a faculty-led debriefing in which the correct interpretation, relevant forensic principles, clinical correlations, and limitations of image-based inference were discussed.

The faculty members conducting the sessions followed a standardized facilitator guide. Faculty, rather than the AI system, remained responsible for all teaching, clarification, interpretation, correction of misconceptions, and final content validation. Allocation was performed at the individual-student level, whereas the educational intervention was delivered in groups. Separate CBL sessions were conducted for the AI-generated image and conventional-image groups. The two groups were taught in separate classrooms during separate sessions. Although the sessions were conducted during the same overall teaching period, students were explicitly instructed not to share the study images or teaching materials with students from the other group to minimize potential contamination. One CBL session was conducted for each selected competency, and all students in the respective study arm attended the applicable sessions. The same faculty members conducted the sessions for both study arms using the same learning objectives, case scenarios, teaching sequence, duration, questioning, and debriefing procedures. As the intervention was delivered in groups, potential within-session correlation between student observations was acknowledged; however, the teaching session was not included as a separate clustering factor in the primary analysis.

The educational intervention was based on the mapping of selected NMC competencies to specific learning objectives, image-based CBL tasks, and assessment domains. The learning objectives progressed from recognition and description of forensic injuries to mechanism-based reasoning, medico-legal application, documentation, and recognition of limitations of image-based assessment (Table 1).

**Table 1:** Mapping of NMC competencies to learning objectives, case-based learning tasks, and assessment domains FM: Forensic Medicine followed by competency number; NMC: National Medical Commission; CBL: case-based learning

NMC competency	Derived learning objective	Image-based CBL task	Assessment domain
FM5.1	Identify and classify common mechanical injuries using morphological features	Observe and identify injury patterns	Recognition, description and classification
FM5.1	Describe injuries using appropriate forensic terminology	Construct a systematic forensic description	Clinical reasoning
FM5.1	Correlate injury morphology with possible mechanisms	Infer plausible mechanisms	Mechanism-based reasoning
FM5.1	Explain medico-legal significance	Determine appropriate conclusions	Medico-legal application
FM14.1	Identify findings requiring documentation	Identify relevant findings from a simulated case	Clinical application
FM14.1	Prepare appropriate medico-legal description	Convert visual findings into report-style documentation	Documentation
FM14.1	Formulate a reasoned medico-legal opinion	Integrate image, history and examination information	Clinical reasoning
FM14.1	Recognize limitations of image-based assessment	Identify additional information required	Critical reasoning

Assessment and outcome measures

Student outcomes were assessed at three predefined time points: T0, T1, and T2.

The primary outcome was clinical reasoning performance. An investigator-developed image-based clinical reasoning assessment consisting of eight case scenarios was used. Each scenario contained four structured questions assessing progressive reasoning from observation and description to interpretation and medico-legal application, producing a maximum score of 32. Responses were evaluated using a predefined scoring rubric developed from the learning objectives and reviewed by five faculty members. The scoring rubric was finalized before assessment of the main study sample. Clinical reasoning responses from all participants were independently scored by two faculty raters who were blinded to intervention-group allocation. The two raters scored all participant responses independently using the same predefined rubric. The rubric awarded marks for accurate identification, appropriate forensic terminology, logical interpretation of morphology, mechanism-based reasoning, appropriate medico-legal application, and recognition of limitations where applicable. The same conceptual domains were assessed at all three time points, while equivalent rather than identical question forms were used where feasible to reduce recall effects. Assessment cases were designed to assess the same learning objectives without directly repeating the teaching cases. Thus, the assessment required students to integrate visual findings with contextual information to interpret morphology, infer plausible mechanisms, apply findings to medico-legal scenarios, and identify the limitations of image-based conclusions, rather than relying on image recognition or recall alone.

The assessment cases were independent of the teaching cases and did not directly repeat the cases or injury patterns used during the intervention; different case presentations were used to minimize familiarity and recall effects.

The instrument was developed de novo for this study based on the study learning objectives and the clinical reasoning processes expected in undergraduate forensic medicine. The construct was operationalized as a progression from recognition and description of visual findings to morphological interpretation, mechanism-based reasoning, medico-legal application, and recognition of the limitations of image-based conclusions. Thus, the assessment was designed to capture applied clinical reasoning rather than image recognition alone. Expert review, pilot testing, and inter-rater reliability assessment provided initial evidence regarding content appropriateness and scoring consistency; these procedures do not by themselves establish construct or criterion-related validity.

Secondary outcomes included knowledge acquisition and retention, student engagement, confidence, and satisfaction. Knowledge was assessed using a 30-item single-best-answer multiple-choice questionnaire developed from the selected CBME competencies. The questions covered mechanical injuries, medico-legal examination and documentation, selected thermal injuries, and selected asphyxial findings. Approximately 60% of the questions were designed at the application or interpretation level rather than simple factual recall. Each correct response received one mark, giving a maximum score of 30.

Student engagement was assessed using a 15-item investigator-developed questionnaire using a five-point Likert scale ranging from strongly disagree to strongly agree. Items assessed cognitive engagement, active participation, attention, perceived relevance, perceived usefulness of visual learning, motivation to participate in case discussion, and willingness to use image-based CBL in future learning. Confidence was assessed using five-point Likert-scale items addressing confidence in identifying forensic findings, describing findings using appropriate terminology, interpreting morphology, applying findings to a medico-legal scenario, and formulating an appropriate conclusion. Overall satisfaction with the learning experience was assessed immediately after the intervention.

The assessment instruments were reviewed by five faculty experts for content relevance, clarity, alignment with the learning objectives, and appropriateness for the academic level. A pilot assessment was performed among 25 students who were not included in the final study sample. Item-level modifications were made before the main study. Internal consistency of the questionnaire domains was assessed using Cronbach's alpha.

The same conceptual domains were assessed at all three time points, while equivalent rather than identical question forms were used where feasible to reduce recall effects. The primary comparison was the difference in clinical reasoning performance between the AI-image and conventional-image groups at T1 after accounting for baseline performance. Retention of clinical reasoning and knowledge was assessed at T2.

The assessment framework included image-based clinical reasoning cases, knowledge-based multiple-choice questions, and structured measures of engagement, confidence, and satisfaction assessed at predefined time points (Table 2).

**Table 2:** Assessment blueprint MCQs: multiple choice questions; T0, baseline assessment before the educational intervention; T1, immediate post-intervention assessment; T2, five- to six-week retention assessment

Assessment measure	Format	Number of items/cases	Maximum score	Primary domain	Assessment time point
Clinical reasoning assessment	Image-based case scenarios	8 cases × 4 questions	32	Recognition, description, interpretation, mechanism-based and medico-legal reasoning	T0, T1, T2
Knowledge assessment	Single-best-answer MCQs	30	30	Knowledge, application and interpretation	T0, T1, T2
Student engagement	5-point Likert questionnaire	15 items	75	Cognitive engagement, participation, attention, relevance and motivation	T1
Confidence	5-point Likert questionnaire	5 items	25	Identification, description, interpretation and medico-legal application	T0, T1
Satisfaction	5-point Likert questionnaire	5 items	25	Perceived educational usefulness and satisfaction	T1

Statistical analysis

Data were entered into IBM SPSS version 26.0 (IBM Corporation, Armonk, NY, USA) and independently checked for completeness and accuracy. Continuous variables were summarized as mean and standard deviation or median and interquartile range, according to the distribution of the data. Categorical variables were summarized as frequencies and percentages.

For the primary outcome, the immediate post-intervention clinical reasoning score was compared between the AI-generated image and conventional-image groups using analysis of covariance (ANCOVA), with the baseline clinical reasoning score included as a covariate. Academic phase was included as an additional factor to account for potential differences between Phase II and Phase III students. The adjusted between-group difference, 95% confidence interval, p-value, and standardized effect size were reported.

For adjusted between-group comparisons, Cohen's d was calculated by dividing the adjusted mean difference by the pooled standard deviation of the corresponding outcome measure.

Changes in clinical reasoning and knowledge scores across T0, T1, and T2 were analyzed using repeated-measures analysis of variance, with intervention group (AI-generated image vs. conventional image) as the between-subjects factor and time (T0, T1, and T2) as the within-subjects factor. Sphericity was assessed using Mauchly's test, and the Greenhouse-Geisser correction was applied when the assumption of sphericity was violated. The group × time interaction was examined to determine whether changes in outcomes over time differed between the two intervention groups.

For the secondary outcomes, post-intervention knowledge and confidence scores were compared between groups using ANCOVA, adjusting for the corresponding baseline score and academic phase. Engagement and satisfaction were assessed only at T1 and were therefore compared without baseline adjustment.

Internal consistency of the investigator-developed Likert-scale instruments was assessed using Cronbach's alpha. The reliability coefficients were 0.835 for the engagement questionnaire, 0.795 for the confidence questionnaire, and 0.88 for the satisfaction questionnaire. Inter-rater reliability for the clinical reasoning assessment was assessed using a two-way random-effects intraclass correlation coefficient (ICC) for absolute agreement (single measures). The ICC was 0.82 (95% CI, 0.785-0.850), indicating good inter-rater reliability.

Missing data were handled according to the requirements of each analysis. No participants had missing baseline (T0) data. Among the 249 allocated students, 22 participants had missing T1 assessments, including nine in the AI-generated image group and 13 in the conventional-image group. A further nine participants completed the T1 assessment but had missing T2 data, including seven in the AI-generated image group and two in the conventional-image group. The primary post-intervention analyses therefore included all 227 participants with available T0 and T1 data, comprising 116 participants in the AI-generated image group and 111 in the conventional-image group. The longitudinal analysis of outcomes across T0, T1, and T2 was performed among the 218 participants with complete data at all three time points, comprising 109 participants in each group. No imputation was performed.

Because the primary ANCOVA already included all participants with available T0 and T1 data irrespective of T2 completion, an additional sensitivity analysis using the same T0/T1 analysis population would not constitute a different analysis.

Clinical reasoning was the prespecified primary outcome. Knowledge, engagement, confidence, and satisfaction were considered secondary outcomes and were analyzed as supportive/exploratory outcomes. No adjustment for multiple comparisons was applied to the secondary outcome analyses. Accordingly, secondary outcome p-values were interpreted cautiously, with emphasis on the estimated between-group differences and 95% confidence intervals (CIs) rather than statistical significance alone. All statistical tests were two-sided, and a p-value of <0.05 was considered statistically significant. Effect sizes and 95% CIs were reported wherever appropriate.

## Results

A total of 249 students were allocated to the study groups, comprising 125 students in the AI-generated image group and 124 students in the conventional-image group. The baseline characteristics of the two groups were similar. There were no statistically significant differences in age, sex distribution, academic phase, baseline clinical reasoning score, baseline knowledge score, or baseline confidence score between the AI-generated image and conventional-image groups (Table 3).

**Table 3:** Baseline characteristics of participants according to the intervention group

Characteristic	AI-generated image group (n = 125)	Conventional-image group (n = 124)	P-value
Age, years, mean ± SD	21.5 ± 1.3	21.3 ± 1.2	0.208
Sex, n (%)			0.852
Male	67 (53.6%)	65 (52.4%)	
Female	58 (46.4%)	59 (47.6%)	
Academic phase, n (%)			0.950
Phase II MBBS	64 (51.2%)	63 (50.8%)	
Phase III MBBS	61 (48.8%)	61 (49.2%)	
Baseline clinical reasoning score (0–32), mean ± SD	14.2 ± 4.1	14.1 ± 3.8	0.842
Baseline knowledge score (0–30), mean ± SD	16.9 ± 3.4	17.1 ± 3.6	0.653
Baseline confidence score (5–25), mean ± SD	16.1 ± 2.9	15.5 ± 2.8	0.098

The AI-generated image group demonstrated significantly better performance in the primary outcome of clinical reasoning. The mean post-intervention clinical reasoning score was 25.3 ± 3.9 in the AI-generated image group compared with 23.1 ± 4.2 in the conventional-image group. After adjustment for baseline clinical reasoning and academic phase, the adjusted between-group difference was 2.4 points (95% CI, 1.4-3.4; F = 22.05; p < 0.001), with a Cohen's d of 0.59 (Table 4).

Secondary analyses showed higher post-intervention scores in the AI-generated image group for knowledge, engagement, confidence, and satisfaction. Knowledge scores were higher in the AI-generated image group than in the conventional-image group (25.1 ± 2.9 vs. 23.7 ± 3.1; adjusted mean difference, 1.3; 95% CI, 0.5-2.1; p = 0.002; Cohen's d = 0.43). Engagement scores were 64.6 ± 6.2 and 62.5 ± 7.2, respectively (adjusted mean difference, 4.7; 95% CI, 2.7-6.7; p < 0.001; Cohen's d = 0.70). Confidence scores were higher in the AI-generated image group (21.4 ± 2.3 vs. 19.6 ± 2.8; adjusted mean difference, 1.6; 95% CI, 0.9-2.3; p < 0.001; Cohen's d = 0.63). Satisfaction scores were 22.8 ± 2.1 and 20.5 ± 2.8, respectively (adjusted mean difference, 2.1; 95% CI, 1.5-2.7; p < 0.001; Cohen's d = 0.85) (Table 4).

**Table 4:** Comparison of primary and secondary outcomes between AI-generated and conventional image-based case-based learning groups Adjusted mean differences represent the AI-generated image group minus the conventional-image group. The primary analysis was adjusted for baseline clinical reasoning score and academic phase. Effect sizes are standardized between-group differences. Clinical reasoning was the prespecified primary outcome.

Outcome	AI	Conventional	Adjusted mean difference	F	P-value	d
Clinical reasoning	25.3 ± 3.9	23.1 ± 4.2	2.4 (1.4–3.4)	22.05 (1,223)	<0.001	0.59
Knowledge	25.1 ± 2.9	23.7 ± 3.1	1.3 (0.5–2.1)	10.1 (1,223)	0.002	0.43
Engagement	64.6 ± 6.2	62.5 ± 7.2	4.7 (2.7–6.7)	21.4 (1,224)	<0.001	0.70
Confidence	21.4 ± 2.3	19.6 ± 2.8	1.6 (0.9–2.3)	19.9 (1,223)	<0.001	0.63
Satisfaction	22.8 ± 2.1	20.5 ± 2.8	2.1 (1.5–2.7)	48.4 (1,224)	<0.001	0.85

Clinical reasoning scores increased from baseline to the immediate post-intervention assessment in both groups, with a greater numerical increase in the AI-generated image group. Among participants with complete longitudinal data, the mean clinical reasoning score increased from 14.2 ± 4.1 at baseline to 25.4 ± 4.0 at T1 in the AI-generated image group, corresponding to a mean increase of 11.2 points. In the conventional-image group, the score increased from 14.1 ± 3.8 to 22.8 ± 4.2, corresponding to an increase of 8.7 points. At the five- to six-week retention assessment, the mean scores remained above baseline in both groups, at 23.2 ± 4.1 in the AI-generated image group and 20.9 ± 4.3 in the conventional-image group. The group × time interaction was statistically significant (F = 18.6, df = 1.82, 393.1; p < 0.001). Knowledge scores showed a similar pattern, with increases from 16.9 ± 3.4 to 25.1 ± 2.9 in the AI-generated image group and from 17.1 ± 3.6 to 23.7 ± 3.1 in the conventional-image group at T1. The group × time interaction for knowledge was also statistically significant (F = 5.7, df = 1.76, 380.2; p = 0.004) (Table 5).

**Table 5:** Changes in clinical reasoning and knowledge scores from baseline to immediate post-intervention and retention assessment Abbreviations: T0, baseline; T1, immediate post-intervention; T2, five- to six-week retention assessment.

Outcome	Group	T0 Baseline, Mean ± SD	T1 Immediate post-intervention, Mean ± SD	T2 5–6 weeks, Mean ± SD	Change T0–T1	Change T0–T2	Group × Time F (df)	P-value
Clinical reasoning	AI-generated image (n=109)	14.2 ± 4.1	25.4 ± 4.0	23.2 ± 4.1	+11.2	+9	18.6 (1.82, 393.1)	<0.001
	Conventional image (n=109)	14.1 ± 3.8	22.8 ± 4.2	20.9 ± 4.3	+8.7	+6.8	—	—
Knowledge	AI-generated image (n=109)	16.9 ± 3.4	25.1 ± 2.9	23.4 ± 3.0	+8.2	+6.5	5.7 (1.76, 380.2)	0.004
	Conventional image (n=109)	17.1 ± 3.6	23.7 ± 3.1	21.8 ± 3.2	+6.6	+4.7	—	—

The assessment instruments demonstrated acceptable content validity and reliability. The scale-level content validity indices ranged from 0.90 to 0.94 across the assessment instruments. The clinical reasoning assessment demonstrated an ICC of 0.82, while the knowledge questionnaire demonstrated a KR-20 coefficient of 0.81. Cronbach's alpha coefficients were 0.835 for engagement, 0.795 for confidence, and 0.88 for satisfaction, indicating satisfactory internal consistency (Table 6).

**Table 6:** Reliability and content validity of the study assessment instruments Abbreviations: S-CVI/Ave, scale-level content validity index based on the average item-level content validity index; ICC, intraclass correlation coefficient; KR-20, Kuder–Richardson Formula 20.

Assessment instrument	No. of items/cases	Content validation	Reliability statistic	Reliability coefficient (CI)
Clinical reasoning assessment	8 cases / 32 marks	Five faculty experts; S-CVI/Ave = 0.93	ICC	0.82 (0.785-0.850)
Knowledge questionnaire	30 MCQs	Five faculty experts; S-CVI/Ave = 0.90	KR-20	0.81 (0.760-0.840)
Engagement questionnaire	15 items	Five faculty experts; S-CVI/Ave = 0.94	Cronbach's α	0.835 (0.790-0.870)
Confidence questionnaire	5 items	Five faculty experts; S-CVI/Ave = 0.92	Cronbach's α	0.795 (0.740-0.840)
Satisfaction questionnaire	5 items	Five faculty experts; S-CVI/Ave = 0.93	Cronbach's α	0.88 (0.850-0.910)

## Discussion

The present study found that students receiving expert-validated AI-generated forensic images as visual triggers within standardized CBL had higher post-intervention clinical reasoning scores than students receiving conventional forensic images. The adjusted between-group difference in the primary outcome was 2.4 points (95% CI, 1.4-3.4; p < 0.001). The corresponding effect size was moderate (Cohen's d = 0.59). Higher post-intervention knowledge, engagement, confidence, and satisfaction scores were also observed in the AI-generated image group. Clinical reasoning and knowledge showed significant group × time interactions across the three assessment points.

The improvement observed in both groups supports the established effectiveness of CBL as an active learning strategy. Cen et al. included eight randomized controlled trials involving 939 medical students and found that CBL significantly improved academic performance and case-analysis ability compared with other teaching methods [3]. Similarly, Lu et al. reported in a recent meta-analysis that CBL combined with problem-based learning significantly improved theoretical knowledge, practical skills, clinical thinking, and learning satisfaction compared with lecture-based learning [4]. The present study is consistent with these findings, as clinical reasoning improved substantially from baseline in both groups. However, the greater improvement in the AI-generated image group suggests that the nature of the visual stimulus may be associated with differences in educational outcomes when incorporated into an already structured case-based framework.

This interpretation is further supported by the randomized study of Weidenbusch et al., in which interactive clinical case discussions produced better clinical reasoning outcomes than paper-based cases, with the advantage remaining evident at delayed assessment [11]. Their findings highlight the importance of active learner engagement and interaction in the development of clinical reasoning. The present study similarly required students to progress from visual observation and description to interpretation, mechanism-based reasoning, and medico-legal application rather than simply identify the injury. Thus, the benefit observed may reflect the interaction between a structured reasoning process and a carefully selected visual stimulus rather than AI generation alone.

The findings are particularly relevant when compared with the recent study by Xie et al., in which AI-generated images were used to teach infectious keratitis to fourth-year medical students [12]. Diagnostic accuracy increased from 42.68% to 71.27% after teaching with AI-generated images, representing a 28.43-percentage-point improvement, and the magnitude of improvement was comparable to that obtained using real medical images and real cases. Our findings extend this evidence in two important ways. First, Xie et al. evaluated diagnostic accuracy, whereas the present study assessed a broader clinical reasoning construct that required students to recognize, describe, interpret, and reason from forensic findings. Second, their intervention compared different teaching modalities, whereas the present study standardized the case content, learning objectives, questioning sequence, faculty facilitation, session duration, and debriefing between groups, with the visual stimulus being the principal intervention difference [12]. The current findings therefore suggest that AI-generated images may have utility beyond pattern recognition and can potentially support more complex image-based reasoning when appropriately integrated into CBL.

The role of visual analysis is also supported by the systematic review by Cerqueira et al., which included 11 studies of Visual Thinking Strategies in medical education [13]. Most studies focused on improving observation or visual diagnostic skills, and several demonstrated significant improvement in observational performance. Although the review noted heterogeneity and methodological limitations among the included studies, it concluded that structured visual analysis can promote deeper observation and clinically relevant interpretation. The present study applies a similar principle to forensic education: the image was not treated as a passive illustration but as a cognitive trigger from which students were required to extract morphological information and progressively construct a clinical and medico-legal interpretation. This may explain why the intervention produced an effect on clinical reasoning rather than only image recognition.

The findings for knowledge acquisition are also consistent with emerging evidence on AI-supported case-based education.

The findings for knowledge acquisition are also consistent with emerging evidence on AI-supported case-based education. Wei et al. synthesized six randomized controlled trials evaluating AI-supported PBL/CBL and found a significant improvement in knowledge acquisition (standardized mean difference (SMD)=0.46; 95% CI, 0.18-0.73; p=0.001) and learner satisfaction, although the overall risk of bias was judged to have some concerns and clinical reasoning could not be quantitatively pooled because of substantial heterogeneity in outcome measures [14]. These findings relate to AI-supported education more broadly rather than specifically to AI-generated images and therefore provide contextual rather than direct evidence for the present intervention.

In the present study, the adjusted difference in knowledge was 1.3 points (95% CI, 0.5-2.1; p=0.002; Cohen's d=0.43), while engagement and satisfaction showed larger effects. The smaller effect on knowledge compared with clinical reasoning may indicate that AI-generated images are particularly useful when learners must integrate visual information with contextual and conceptual knowledge rather than simply acquire factual information.

The higher post-intervention engagement, confidence, and satisfaction scores observed in the AI-generated image group are also consistent with the broader literature on AI-supported learning. The pooled satisfaction benefit reported by Wei et al. supports the finding of greater satisfaction in the present study, while the larger effect observed for satisfaction (Cohen's d=0.85) may partly reflect the novelty and visual flexibility of generative AI [14]. However, the present findings should not be interpreted as evidence that novelty alone produced the educational benefit because the increase in satisfaction occurred alongside objectively measured improvements in clinical reasoning and knowledge. Similarly, the increase in confidence should be interpreted cautiously because confidence is subjective; its educational significance is stronger when accompanied by demonstrated improvement in performance, as observed in this study.

Because multiple secondary outcomes were examined without adjustment for multiplicity, these findings should be interpreted cautiously as supportive and exploratory rather than as independent confirmatory evidence.

The retention findings provide an additional important contribution. Clinical reasoning remained substantially above baseline at five to six weeks in both groups, but the AI-generated image group maintained a greater gain. Knowledge showed a similar pattern, with a significant group × time interaction. This finding is consistent with the delayed benefit reported by Weidenbusch et al. following interactive case discussion, although their follow-up period was shorter [11]. In contrast, Çiçek et al. found no significant difference between ChatGPT-generated and expert-written feedback in overall clinical reasoning performance immediately after intervention or at 10 days [15]. The difference may relate to the nature of the AI intervention. In their study, AI generated feedback on clinical reasoning questions, whereas in the present study AI was restricted to generating the visual stimulus and students were required to perform the reasoning independently under faculty supervision. This distinction is important because it suggests that using AI to create a learning stimulus may support reasoning differently from using AI to perform part of the reasoning process for the learner.

A possible explanation for the observed benefit is the ability of generative AI to produce visual material closely aligned with predefined learning objectives. In the present study, images were generated according to specified forensic findings, anatomical locations, and clinical contexts, and students were subsequently exposed to standardized reasoning prompts. This permits educators to generate multiple examples of specific injury patterns and potentially provide greater variation than a limited collection of conventional photographs. Such variation may facilitate repeated exposure to morphological features while maintaining consistency in the intended learning objective. Nevertheless, the educational value should be attributed to the AI-supported instructional design, rather than to image generation in isolation.

The validation process in the present study is particularly important in this context. Of 100 AI-generated images, only 62 met the predefined expert-validation criterion and were retained for teaching. Thus, 38% of the generated images did not meet the predefined validation criterion, highlighting that AI-generated images require substantial human screening and curation before educational use. The recent systematic review by Alon et al. provides strong support for this concern. Among 36 empirical studies evaluating AI-generated images in medical education, clinical-fidelity limitations were reported in 17 studies (47.2%), while demographic representational bias was also frequently observed [16]. The authors emphasized that high visual plausibility can conceal clinically incorrect representations and recommended expert curation and governance before educational use. This concern is directly relevant to forensic medicine, where subtle morphological inaccuracies could lead students to form incorrect assumptions regarding injury type, mechanism, or medico-legal significance.

The present study therefore supports a human-in-the-loop approach to generative AI in medical education. AI was used to generate the visual material, but faculty retained responsibility for validation, case interpretation, questioning, correction of misconceptions, and debriefing. This approach may be particularly appropriate for forensic medicine because the educational objective extends beyond recognition of an image to interpretation of injury morphology within a clinical and medico-legal context. AI-generated images should therefore be considered supplementary resources rather than replacements for authentic clinical material or expert instruction.

The study has several strengths. The study included 249 allocated students, with 227 contributing to the primary T0-T1 analysis and 218 contributing to the complete longitudinal analysis. The groups were similar at baseline with respect to demographic variables, academic phase, clinical reasoning, knowledge, and confidence. The intervention and control groups received the same cases, learning objectives, questioning sequence, faculty facilitation, duration, and debriefing, thereby reducing variation in instructional delivery. The primary outcome was an objective assessment of clinical reasoning rather than learner satisfaction alone, and the inclusion of a five-to-six-week retention assessment provided information regarding persistence of learning. Expert validation of the AI-generated images and acceptable reliability of the assessment instruments further strengthen the study.

Several limitations should be considered. The study was conducted at a single institution among Phase II and III MBBS students, limiting generalizability. The non-randomized allocation may have introduced selection bias, including differences in unmeasured characteristics despite similar measured baseline variables. The intervention focused on forensic injury interpretation and may not generalize to other disciplines. The clinical reasoning assessment and questionnaires were investigator-developed, although they underwent expert review and demonstrated acceptable reliability. The visual stimuli were matched for forensic finding, anatomical site, learning objective, and educational purpose; however, formal matching for visual complexity, realism, image quality, diagnostic salience, and difficulty was not performed. Therefore, the observed differences cannot be attributed exclusively to AI generation. Blinding was not feasible, allowing possible expectancy effects. Although retention was assessed at five to six weeks, longer-term retention and transfer to authentic clinical or medico-legal practice were not evaluated. Finally, findings may vary with different AI models, prompting strategies, or image-generation platforms. Accordingly, the findings should be considered specific to the study setting, learner population, faculty team, and AI-generation workflow and should not be assumed to generalize to other educational or clinical contexts.

Overall, the findings suggest that AI-generated images can provide educational value when embedded within a structured pedagogical framework rather than introduced as an independent technology. Evidence from CBL studies supports the underlying active-learning approach, while the visual-learning literature supports the use of structured image analysis, and emerging AI studies suggest that generative images can support diagnostic learning. The present study adds to this literature by demonstrating an association between expert-validated AI-generated forensic images and improved higher-order clinical reasoning, with benefits extending to knowledge, engagement, confidence, satisfaction, and short-term retention. Future multicenter studies should evaluate whether these findings are reproducible across institutions and specialties, compare different AI-generation platforms, examine longer-term retention, and determine whether improvements in image-based reasoning translate into better performance in practical examinations and authentic medico-legal decision-making.

## Conclusions

This study found that the use of expert-validated AI-generated forensic images as visual triggers within standardized CBL was associated with greater improvement in measured clinical reasoning among Phase II and Phase III MBBS students. Compared with conventional forensic images, the AI-generated image group showed significantly greater improvement in clinical reasoning, together with higher knowledge, engagement, confidence, and satisfaction scores. Clinical reasoning and knowledge scores remained above baseline at five to six weeks in both groups, with a significant group-by-time interaction. These findings indicate short-term retention within the study period but do not establish durable educational retention or transfer to clinical practice.

Importantly, the observed educational differences occurred within an intervention that integrated AI-generated images with standardized cases, structured questioning, faculty facilitation, and debriefing and therefore cannot be attributed to AI use alone. Expert validation remains essential because AI-generated clinical images may contain inaccuracies or unrealistic visual features. Accordingly, AI-generated images should be regarded as an adjunct to, rather than a replacement for, faculty-guided clinical reasoning and conventional educational resources. Multicenter studies with longer follow-up and assessment of transfer to authentic clinical and medico-legal decision-making are warranted to determine the broader educational effectiveness and applicability of this approach.

## Appendices

Representative AI-generated prompt for Figure 2

Generate a medically and forensically realistic clinical photograph of an adult female showing multiple superficial, linear abrasions on the lateral aspect of the neck. The injuries should be clearly visible and morphologically consistent with linear abrasions, with realistic superficial epidermal disruption, mild erythema, and subtle dried blood where appropriate. The anatomical appearance of the neck, skin texture, surrounding structures, and injury morphology should be realistic and suitable for undergraduate forensic medicine education. The image should resemble a clinical forensic photograph rather than an artistic illustration. Use a neutral clinical presentation with adequate lighting and clear visualization of the injury. Do not include diagnostic labels, arrows, text, captions, or other annotations. The image should allow students to independently observe and describe the injury morphology and subsequently reason about possible mechanisms and medico-legal significance.
